# Bibliographical

**Published:** 1856-03

**Authors:** 


### BIBLIOGRAPHICAL.

*Ranking's Abstract.*—We have received from Messrs. Lindsay & Blackiston, the publishers, Philadelphia, Ranking's Abstract from July to December, 1855—being a practical and analytical digest of the contents of the principal British, American and Continental medical works published during the six months, embraced, together with a series of Critical Reports on the progress of medicine and collateral sciences, for the same period—this number containing references to forty-nine periodicals, &c., of British, American, French, German and Italian issue. It is unquestionably a work of great value; and one of which, we think, every practitioner would do well to avail himself—the cheap, accessible, and condensed form in which the most valuable and practical results, determined by the most prominent men of the profession, are presented, commend it, especially, to the active physician, who cannot afford the time to wade through the almost interminable mass of medical productions, and which are, indeed, accessible to but few. The work is furnished at $2 per annum, in half yearly numbers.

*Principles of Human Physiology*—With their chief applications to Psychology, Pathology, Therapeutics, Hygeine, and Forensic Medicine, by William B. Carpenter, M. D., F. R. S., F. G. S., Examiner in Physiology and Comparative Anatomy in the University of London; Professor of Medical Jurisprudence in University College; President of the Microscpical Society of London, &c., &c.—A New American from the last London edition—with two hundred and sixty-one illustrations—edited with additions, by Francis Gurney Smith, M. D., Professor of the Institutes of Medicine in the Medical Department of Pennsylvania College, &c. Philadelphia: Blanchard & Lea, 1855. Received from the publishers.

The above is the title of what is emphatically *the* great work on Physiology; and we are conscious that it would be a useless effort to attempt to add any thing to the reputation of this invaluable work, and can only say to all with whom our opinion has any influence, that it is our *authority*, and they will certainly be deprived of a treasure, if they fail to have it in their libraries.

The present volume contains nearly two hundred pages less than the American edition of 1853, being the result of a rearrangement of the author's works on the comprehensive science of Physiology; and by the way of giving a better idea of the exact character of the present edition than we could present, that portion of the author's preface which has an especial reference to the alteration of the character of the work, is here given. In accordance with the plan adopted, he remarks: "The second, third, and fifth chapters of the last edition of this work, which included a summary of Animal Chemistry, and of the Structure and Actions of the Animal Tissues, amounting in all to about 240 pages, have been omitted from the present. On the other hand, additions have been made to the amount of about 70 pages; and these by no means constitute the whole of the new matter introduced, since many portions have been re-written, with little or no increase of bulk. It has been the author's desire, on this as on former occasions, that his treatise should represent his present convictions and opinions, as completely as if it were making its appearance for the first time; and he has accordingly subjected every part of it to a revision not less careful than that which he would have bestowed upon it, had it less recently passed under a similar scrutiny. Although the minor results of this revision, which are scattered through almost every part of the volume, would not be apparent save on a searching comparison, yet he trusts that they will be found to have increased the utility of the work; those of more importance, however, he deems it well now to particularize.

"In the chapters which treat of the several Organic Functions, many important additions have been derived from the admirable work of MM. Bidder and Schmidt, 'Die Verdauungssafte und der Stoffwechsel,' which contains the results of those elaborate researches on Digestion, Respiration, Secretion, and the Metamorposis of Tissue, which they have carried on for several successive years in the Dorpat Laboratory. It may be thought, perhaps, by such as are conversant with this work, that the author has not made sufficient use of the vast body of information which it supplies; but he must be permitted to remark, in the first place, that so many of the statements of these able experimenters are in direct contradiction to those of others who had previously stood in good repute, as well as to generally-accepted Physiological doctrines, that it is yet doubtful on which side the truth lies; and, secondly, that even where their facts are not disputed, there is often so much doubt, respecting the right interpretation of them, and more especially in regard to their applicability to man, that he has scarcely judged it expedient to admit such into a treatise which especially aims at embodying the *certainties* of Physiological science.

"The portions of chapter iv. which relate to the Glandulæ of the Absorbent System, and to the Vascular Glands, have been almost entirely re-written, in accordance with the improved knowledge of these bodies which has been recently attained, through the labors of various histological Anatomists and experimental Physiologists, especially Brucke, Kolliker, and H. Gray.

"The part of chapter ix. in which the Minute Anatomy and the Physiology of the Liver are discussed, has been brought into accordance, on most points, with the views entertained by Prof. Kolliker as to its structure, and with those of Dr. C. Handfield Jones in regard to its actions: the author being now convinced that the account of this organ given by Dr. Leidy and by Prof. Retzius, to which he had formerly seen reason to assent, is based on a wrong interpretation of the appearances presented; and that the liver really unites, as well structurally as functionally, the essential characters of a Vascular or Assimilating Gland, with those of an Excretory Gland.

"In chapter xi., on the Functions of the Cerebro-Spinal Nervous System, the author has again seen reason to introduce very considerable modifications; these having reference for the most part, however, rather to the order of *succession* of the subjects, than to the *opinions* previously put forth—the latter, in fact, having received most satisfactory confirmation, alike from the accordance which has been expressed with them by many highly-competent judges, from the author's own more matured reflections, and from certain occurrences of public notoriety which have afforded most remarkable exemplifications of them. It would have been scarcely possible, in fact, to conceive of any more apposite and convincing proof of that independent automatic activity of the Cerebrum, and of its involuntary influence in producing muscular contraction, which the author had formularized in the doctrine of 'Ideo-Motor action,' than that which was afforded by the Epidemic of 'Table-turning' and 'Table-talking,' which, originating in the United States, began to spread through Europe just at the epoch of the publication of the former edition, in which that doctrine was first distinctly developed. And some of the rarer phenomena of that Epidemic also afforded interesting illustrations of his doctrine of 'Unconscious Cerebration;' the validity of which has been admitted by many eminent Psychologists, who have no leaning whatever to what is commonly termed 'Materialism.' Not among the least valuable of the testimonies to the general correctness of the author's views, as to the relation of the Will to the Automatic operations of the Cerebrum, are those which he has received from individuals practically conversant with various departments of education (especially schools for the reformation of juvenile delinquents,) and with the treatment of Insanity. In recasting, as a separate section of this chapter, all that relates to 'The Mind and its Operations,' the author has derived many valuable suggestions and much assistance, in regard to the 'Perceptive and Intuitional Consciousness,' from the valuable 'Elements of Psychology' of his friend Mr. J. D. Morell; whilst, for the extension of his notion of those states of feeling which constitute the essence of emotions, from that of mere *pleasure* and *pain* to which he had previously limited them, to more varied forms of 'Emotional Sensibility,' as well as for the suggestion of that very appropriate term, he is indebted to his friend Dr. Daniel Noble.* The whole of this section has passed under the revision of the Rev. W. Thomson (Fellow and Tutor of Queen's College, Oxford,) the author of the well-known 'Outline of the Necessary Laws of Thought;' for whose kindness in undertaking this labor in behalf of one almost a stranger to him, the author has great pleasure in making this acknowledgment.

"In chapter xii. (part of the first section of which has been

---

* See his "Elements of Psychological Medicine," 2d edit., p. 56.—This little work, the author (though not according in everything it contains) would strongly recommend to all who are entering on the practice of their profession.

transferred to what seemed its more appropriate place in the last-mentioned division,) the section on Vision has received several additions and modifications; the most important of which are derived from the researches of H. Muller on the Structure of the Retina, from the enquiries of Prof. Wheatstone into various points in the Physiology of Binocular Vision, and from the curious investigations made by Dr. Sierre in regard to the subjective phenomena produced by pressure on the Eyeball.

"From chapter xiii., also, the section 'On the Influence of Expectant Attention on Muscular Movements' has been for the most part removed into chapter xi., where it completed the doctrine of Ideo-Motor action; whilst the small portion relating to the movements of the Organic Muscles has been transferred to chapter xv. To this last chapter has also been removed the account of the structure and relations of the Sympathetic System; so that it now embraces a summary of all the principal modes in which the Nervous System, or the Mind through its agency, affects the Organic Functions.

"Various improvements, scarcely worth here particularizing, have been made in chapter xvi., on the Generative Function; the most important additions being a summary of Dr. Dalton's researches on the distinctions between the Corpus Luteum of simple Menstruation and that of Pregnancy, and a notice of certain curious circumstances attending the transmission of Parental characters to the offspring, which have a direct bearing on the question of Marriage of near Relations.

"Chapter xviii., 'On the Modes of Vital Activity Characteristic of different Ages,' has been almost entirely written specially for this edition; the subject, which had been only touched on incidentally in the preceding, appearing to the author to deserve, under every point of view, a more express consideration.

"The entire number of wood engravings has necessarily undergone some reduction, owing to the transference of no fewer than 82, which illustrated the structure of the Primary Tissues, to the 'General Physiology.' But as many as 46 new ones have been introduced, in addition to those which previously illustrated the subjects treated of in the present volume; these having been for the most part drawn from the 'Mikroskopische Anatomie' of Prof. Kolliker, and from the new edition of Prof. Wagner's 'Icones Physiologicæ' now being brought out under the able superintendence of Prof. Ecker."

We have also received from Messrs. D. Appleton & Co., 346 and 348 Broadway, New York, "The Chemistry of Common Life." By Jas F. Johnston, M. A., F. R. S., F. G. S., &c. &c., Author of Lectures on Agricultural Chemistry and Geology, &c., which we can confidently recommend to all who would understand the "relations of human life and health to the air we breath and the water we drink—the soil we cultivate and the plant we rear, as the source from which the ehief sustenance of all life is obtained—the bread we eat and the beef we cook, as the representatives of the two grand divisions of human food—the beverage we infuse, from, which so much of the comfort of modern life, both savage and civilized is derived—the sweets we extract, the history of which presents so striking an illustration of the economical value of chemical science—the liquors we ferment, so different from the sweets in their action on the system, and yet so closely connected with them in chemical history—the narcotics we indulge in, as presenting us with an aspect ot the human constitution which both, chemically and physically, is more mysterious and wonderful than any other we are yet acquainted with—the odors we enjoy and the smells we dislike; the former because of the beautiful illustration they present of the recent progress of organic chemistry in its relations to the comforts of common life, and the latter because of their intimate connection with our most important sanitary arrangements—what we breathe for and why we digest, as relating to functions of the body at once the most important to life, and the most purely chemical in their nature—the body we cherish, as presenting many striking phenomena, and performing many interesting chemical functions not touched upon in the discussion of the preceding topics—and lastly, the circulation of matter, as exhibiting in one view, the end, purpose and method of all the changes in the natural body, in organic nature, and in the mineral kingdom, which are connected with, and determine the existence of life."

